# Some Points Concerning Sanguis Bovinus Exsiccatus (Desiccated Blood)

**Published:** 1884-03-20

**Authors:** F. E. Stewart

**Affiliations:** Manchester, Kentucky


### SOME POINTS CONCERNING SANGUIS BOVINUS EXSICCATUS (DESICCATED BLOOD).

BY F. E. STEWART, M.D., PH. G.,
Of Manchester, Kentucky.

I see that the fifteenth edition of the United States Dispensatory, recently published, gives me credit for the introduction of sanguis bovinus exsiccatus (desiccated blood), as a method of administering iron. As that is only one of its uses, and by no means the principal one, I beg leave to call attention to the following brief summary of what I consider to be the true position of this valuable agent in therapeutics.

Sanguis bovinus exsiccatus is a concentrated nutritive tonic prepared directly from the blood of bullocks, by simply drying it at a low temperature, to prevent the albumen from coagulating by the heat, and in shallow pans. The product, when so prepared, is in the form of thin scales somewhat resembling the scaly salts of the pharmacopœia. The blood may be prevented from clotting by stirring it before drying, and removing the small portion of fibrin that attaches itself to the stirring rod, or by adding a small portion of acid to the blood before submitting it to the desiccating process.

The article thus prepared contains all the elements of the blood, and in a form for easy assimilation; but, more than this, it contains potentially all that vast amount of energy required to raise blood from the plane of elements and of chemicel compounds to that of animal life. It is, therefore, not only an agent for building and repairing tissues, but is, at the same time, a fuel, and its combustion in the system is capable of generating a large amount of vital energy for carrying on the vital processes.

Furthermore, this article contains iron in that peculiar combination found in the blood, and this form, so far as known, can only be manufactured by vital chemistry. The important relation that iron bears to the health of the red blood corpuscle, and the special relations borne by the red corpuscle to respiration and to the nervous system, are well known, and, together with the facts already stated, will probably account for the great benefit following the administration of desiccated blood, even in quantities too small for the credit to be entirely due to the albumen which it contains.

*Indications for use.*—Desiccated blood has been used with benefit in wasting diseases; in various cachexia, such as scrofula, consumption and syphilis; in anæmia and chlorosis; in atonic dyspepsia; after hemorrhages and exhaustive discharges; in the height of the fever process to supply the combustion of tissues incident thereto, and to forestall a prostrated condition endangering life from exhaustion; in convalescence from fevers; and as a "building-up" measure in surgical cases before operations, and after operations to hasten the reparative process. It is indicated in all cases where it is desirable to give iron, but more especially whenever the problem is to give food.

If quickly dried, this product is odorless, and almost entirely devoid of unpleasant taste. It may be administered either by the mouth or by the rectum; preferably, however, by the former method. When administered by the mouth, it may be prescribed in combination with glycerine, with glycerine and brandy, or simply in solution with water. In either case the blood should be first dissolved in water. A very good formula, suggested to me by Dr. Andrew H. Smith, of New York, is the following:

℞ Sang. bov. exsiccat.......... ..................ʒ vj,
Aquæ....................................fl. ℥ iv,
Misce ft. sol.
Adde spts. vin. gal. et glycerinæ ana............℥ j.
S.—Tablespoonful three or four times daily.

In fever cases, when the temperature is high, I am in the habit of giving a teaspoonful, or more. as the stomach will bear it, every hour, carefully watching the dejections to see that it is all assimilated; and its use in this manner has been followed, in a number of cases, by the most gratifying results. In some cases, however, it is not assimilated, and stains the dejections black. In such instances, the addition of pepsin will usually remove the difficulty. As a disguise to the taste, I have been in the habit of employing some of the essential oils, a few drops of which will answer the requirements.

With regard to the use of blood per rectum, I have found that, in some cases it is readily absorbed, and in other cases absorption does not take place. The reason for this I have never been able to ascertain. Pepsin should always be added to the solution when difficulty of this kind is experienced.

A point or two with regard to making a solution of desiccated blood. When preparing an aqueous solution, *never pour the water on the scales; always pour the scales in the water.* This point cannot be too strongly insisted upon. Then stir with a glass rod or spatula until dissolved, taking care to prevent the agglomeration of the scales into a mass. Again, always use *cold* water in forming the solution. Blood contains a large proportion of albumen. In fact, the dry blood is almost all albumen, and albumen coagulates at a temperature of 160° F. There is about as much sense in attempting to dissolve dry blood in hot water as to attempt the solution of an egg in the same medium. In either case the albumen coagulates and becomes hard. If a little more attention were paid to this well-known fact, we would hear less of those displays of stupid ignorance on the part of certain individuals, who have attempted to dissolve desiccated blood in boiling water.

For rectal use, the solution should be gently warmed before injecting, taking care not to raise it to a sufficient temperature to coagulate the albumen.—*Med. and Surg. Rep.*

---

**In** treating an aneurism Dupuytren ligatured the fourth cervical nerve, and Lister, in a similar case, after a considerable time, performed a like operation on the lower cord of the brachial plexus.

